# My Personal Tribute to Dr. Mary Ellen Avery

**DOI:** 10.3389/fped.2014.00023

**Published:** 2014-03-24

**Authors:** Ilene R. S. Sosenko

**Affiliations:** ^1^Division of Neonatology, Department of Pediatrics, University of Miami Miller School of Medicine, Miami, FL, USA

**Keywords:** neonatology, research, IDM, mentor, Mary Ellen Avery

Because my husband was planning to pursue a fellowship in adolescent medicine at Boston Children’s Hospital, I was set to interview there in July of 1974 for a third year of pediatric training. I was just starting my second year of pediatrics in Los Angeles and had been focusing more on the mechanics of completing pediatric training than on choosing a fellowship. Dr. Mary Ellen Avery had just become Children’s Physician-in-Chief. In fact, she was still finding her way about the place, both physically and professionally. It was my second interview, the first resulting in less than positive news about the possibility of my obtaining a senior pediatric residency for the coming year. She did not let me be discouraged, made it known that she would welcome me there, and suggested I could pursue the start of a fellowship before completing my full pediatric training. What type of fellowship? I certainly hadn’t spent much time contemplating this. How about neonatology, she suggested, and immediately arranged for me to see Dr. Bill Taeusch, newly appointed Neonatology Division Director. So serendipity, and more importantly, Dr. Mel Avery are responsible for my career in neonatology: I joined the first official JPN fellows’ group that started training in July of 1975.

Dr. Avery was a pioneering researcher in the field of neonatology and a great research motivator as well. She had overseen a recent publication associating an increased risk of hyaline membrane disease in infants of diabetic mothers (1). She “shepherded” me into examining this further, thus leading me both to the delivery room to collect infants’ cord blood for C-peptide measurements (2) and into the animal lab with alloxan-diabetic pregnant rabbits and the lung development of their offspring (3–7). I remember her delight when I presented my findings at research meetings and had my first peer-reviewed publications in the *New England Journal of Medicine* (2) and *Journal of Applied Physiology* (3). In fact, when I moved on to University of Miami, I continued the line of research looking at lung development in the animal model of the IDM, this time examining pulmonary antioxidant enzyme development in offspring of streptozotocin-treated rats (8).

She opened her heart to me not just professionally but socially and personally as well. I remember a cozy Thanksgiving evening when just she, my husband, and I sat by the fire in her Wellesley condo and dined on delicious leftovers from the day. She hosted the two of us to a very special Boston evening: a “double date” with Dr. Fred Rosen for dinner at the Harvard Club and then to hear the Boston Symphony. When I was about to have my first child, she was glowing when I came to her with the news. She wanted to make sure I would be nursing him which of course I was. When he was born, she presented us with a beautifully framed print of a mother rabbit and her pups (how appropriate!) which we hung above his changing table. And once I had successfully established nursing and returned to work, she invited me to write an article with her on the benefits of breast feeding for the Harvard Medical School Health Letter (9).

My respect and admiration for Dr. Mary Ellen Avery are without bounds. There is no doubt that Mel brought me into neonatology, fostered my career, and opened her intellect and heart to me many years ago. For this I am eternally grateful.
